# Toward a Consistent Pattern of Ecosystem Theories

**DOI:** 10.1100/tsw.2001.15

**Published:** 2001-04-04

**Authors:** Sven Erik Jorgensen

**Affiliations:** Institute A, Section Environmental Chemistry, University Park 2, 2100 Copenhagen Ø, Denmark

## Abstract

Several ecosystem theories have been presented during the last 2–3 decades. Lately it has been possible to unite these theories into a consistent pattern. Intensive discussions at meetings have been the key to the formation of this pattern of ecosystem theories. Another driving factor has been the reformulation of E.P. Odum’s attributes1 into three growth forms: growth of the physical structure (the biomass), growth of the network (more cycling, more linkages), and growth of information. An analysis of the growth forms2 shows that through-flow (power), ascendancy, and exergy storage are all increasing as a consequence of all three types of growth. However, exergy dissipation, entropy production, specific exergy (exergy/biomass), retention time, and the ratio indirect/direct effect only increases or decreases with one or two of the growth forms.

## INTRODUCTION

Several ecosystem theories have been presented in the scientific literature during the last 2–3 decades. At first glance they look very different and seem to be inconsistent, but further examination reveals[3] that they are not so different and that it should be possible to unite them in a consistent pattern. This has been the general belief among system ecologists for the last 3–4 years, but as a result of discussions at two meetings last year (one in Porto Venere, Italy in late May and one in Copenhagen in early June, in conjunction with the ASLO meeting), it can now be concluded that a consistent pattern of ecosystem theories has been formed. Several system ecologists (personal communication with H.T. Odum, B.P. Patten, R. Ulanowicz, B. Fath, James Kay, S. Ulgiati, M. Straskraba, and S.N. Nielsen) agreed on the pattern presented in this paper, as a working basis for further development in system ecology. This is of utmost importance for the progress in system ecology, because with a theory in hand, it will be possible to explain many empirical rules that are published in ecology and applied ecology which again explain many ecological observations. We can, with other words, attain the same theoretical basis that characterizes physics: a few basic laws, which can be used to deduce more specific rules, that explain observations. It has therefore also been agreed that one of the important goals in system ecology in the coming years is to demonstrate (prove) the links between ecological rules and ecological laws, i.e., to construct a theoretical skeleton in system ecology.

The presented theories seemed very inconsistent and chaotic 10–15 years ago. How could E.P. Odum's[1] attributes, Lotka's[4] and H.T. Odum's[5] maximum power, Ulanowicz's[6] ascendency, Patten's[7] indirect effect, Kay and Schneider's[8] maximum exergy degradation (see also Kay[9]), the maximum exergy principle[3,10,11,12,13], and Prigogine's[14,15] and Mauersberger's[16,17] minimum entropy dissipation (supported by Johnson[18,19]) be valid at the same time? Exergy is defined as the maximum amount of work the system can perform when it is brought into thermodynamic equilibrium with its environment. The slightly different definition shown in Fig. 1, where the temperature and the pressure are the same for the system and its environment, has mostly been applied in ecological context.

Everyone insisted that his version of a law for ecosystem development was right, and all the other versions were wrong. New results and an open discussion among the contributing scientists have led to a formation of a pattern, where all the theories contribute to the total picture of ecosystem development.

This paper reviews these new results, and the resulting pattern which may be utilized in the coming year to make significant progresses in system ecology.

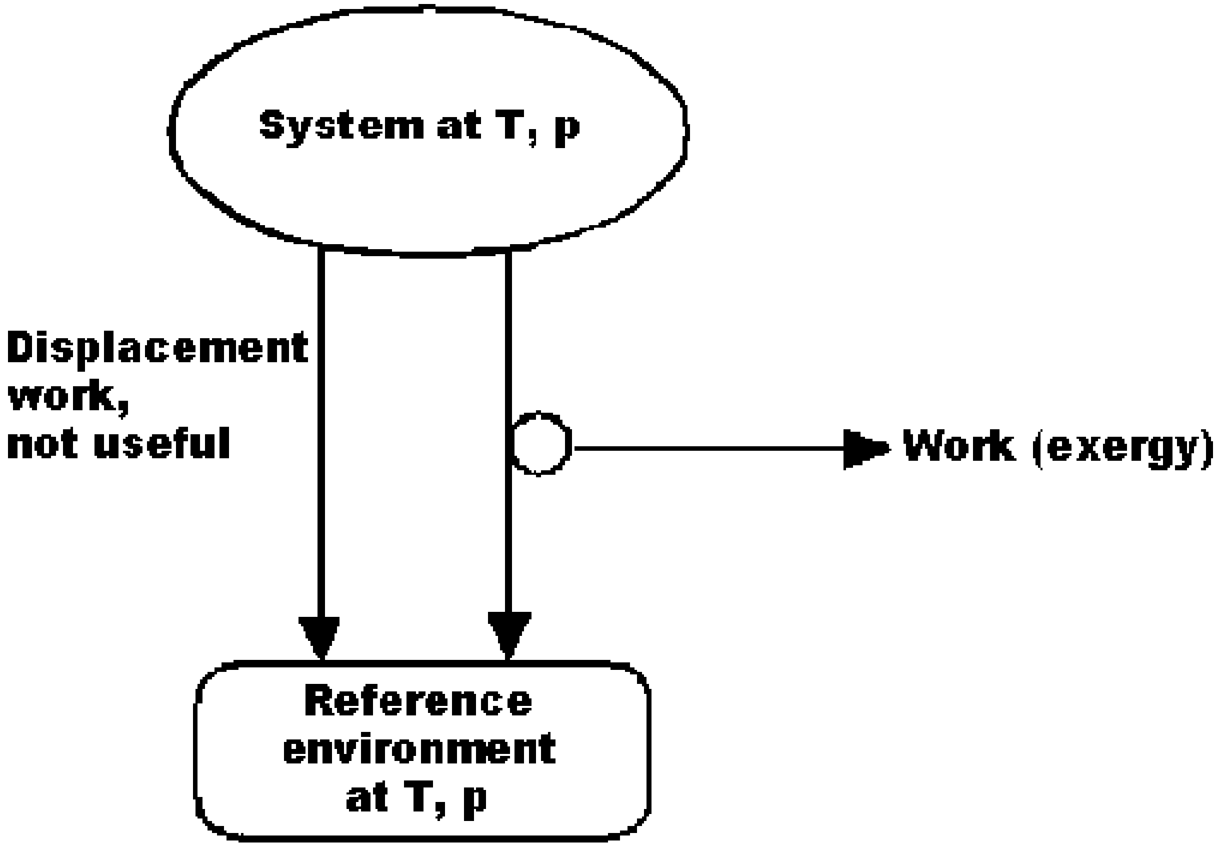

**FIGURE 1.** Illustration of the concept of exergy used to compute the exergy index for an ecological model. Temperature and pressure are the same for both the system and the reference state, which implies that only by the difference in chemical potentials ($\mu_1 - \mu_0$) can work be done.

## THE DEVELOPMENT OF A PATTERN OF ECOSYSTEM THEORIES

The first contribution to a clear pattern of the various ecosystem theories came from network approach used often by Patten[20]. Patten and Fath[21] have shown, by a mathematical analysis of networks in steady state, that the sum of through-flows in a network (which is maximum power) is determined by the input and the cycling within the network. An example of this would be an average annual situation in an ecosystem with close to balanced inputs and outputs for all components in the network. The input from the captured solar radiation is determined by the structure of the system (i.e., the stored exergy and the biomass). Furthermore, more structure means that more maintenance is needed and therefore more exergy must be dissipated for greater input. Cycling on the other hand means that the same energy (exergy) is better utilized in the system, and therefore more biomass (exergy) can be formed without increase of the inputs. It has been shown previously that more cycling means increased ratio of indirect to direct effects, while increased input does not change the ratio of indirect to direct effects[3].

Fath et al.[22] used these results to determine the development of various variables used as goal functions (exergy, power, entropy, etc.). An ecosystem is of course not setting goals, but a goal function is used to describe the direction of development an ecosystem will take in an ecological model. Their results can be summarized as follows:

1. Increased inputs (more solar radiation is captured) mean more biomass, more exergy stored, more exergy degraded. Therefore there is also higher entropy dissipation, more through-flow (power), and increased ascendency. However, there is no change in the ratio indirect/direct effect or in the retention time for the energy in the system = total exergy/input exergy per unit of time.
2. Increased cycling implies more biomass, more exergy stored, more through-flow, increased ascendency, increased ratio indirect/direct effect, and increased retention but no change in exergy degradation.

Almost simultaneously Jørgensen et al.[23] published a paper which claims that development of ecosystems can be described by three growth forms that are consistent with the description by E.P. Odum[1]:

I. Growth of physical structure (biomass) which is able to capture more of the incoming energy in the form of solar radiation, but also requires more energy for maintenance (respiration and evaporation).
II. Growth of network, which means more cycling of energy and matter.
III. Growth of information (more developed plants and animals with more genes), from r-strategists to K-strategists, which waste less energy but also usually carry more information.

These three growth forms are consistent with E.P. Odum's attributes that describe changes in an ecosystem associated with development from the early stage to the mature stage. Eight of the most applied attributes are shown below:

1. Ecosystem biomass (physical structure) increases.
2. More feedback loops (including recycling of energy and matter) are built.
3. Respiration increases.
4. Respiration relative to biomass decreases.
5. Bigger animals and plants (trees) become more dominant.
6. The specific entropy production (relative to biomass) decreases.
7. The total entropy production will first increase and then stabilise on approximately the same level.
8. The amount of information increases (more species, species with more genes, the biochemistry becomes more diverse).

Growth form I covers attributes 1, 3, and 7. Growth form II covers 2 and 6, and growth form III covers the attributes 4, 5, 7, and 8.

In the same paper[2] Fig. 2 was presented to illustrate the concomitant development of ecosystems, exergy captured

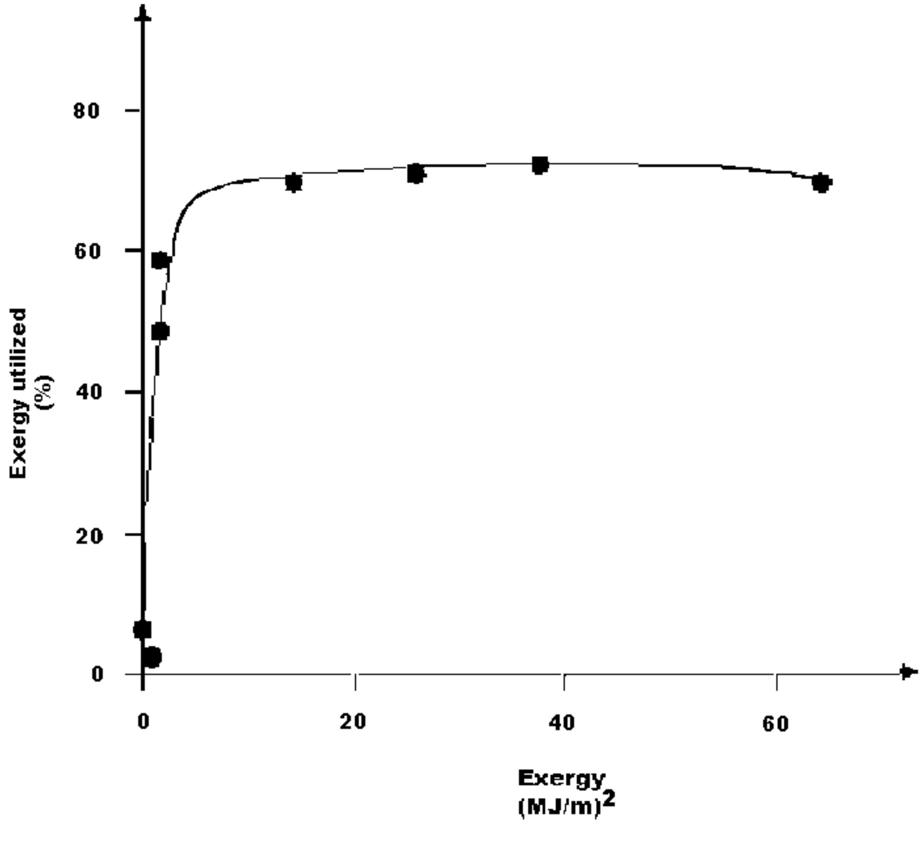

**FIGURE 2.** The exergy captured (data are from Kay and Schneider[8], unit: $J/m^2$ 24 h) is plotted vs. the exergy stored (unit $J/m^2$ or $J/m^3$), calculated from characteristic compositions of the focal 8 ecosystems. The numbers from Table 1 are applied to construct this plot.

**TABLE 1**
**Exergy Utilization and Exergy Storage**

| Ecosystem | % Exergy utilization | Exergy storage kJ/m$^2$ |
|---|---|---|
| Quarry | 6 | 0 |
| Desert | 2 | 73 |
| Clear cut | 49 | 594 |
| Grassland | 59 | 940 |
| Fir plantation | 70 | 12700 |
| Natural forest | 71 | 26000 |
| Old deciduous forest | 72 | 38000 |
| Tropical rain forest | 70 | 64000 |

(most of that being degraded), and exergy stored (biomass, structure, information). The points in the figures correspond to ecosystems at different stages of development (see Table 1, which shows the values). Debeljak[24] has obtained the same shape of the curve when he determines exergy captured and exergy stored in managed forest and virgin forest at different stages of development. Jørgensen et al.[23] have set up the relationship between the three growth forms and the various goal functions, mentioned above. They conclude that increase of exergy storage and through-flow describe ecosystem development based on all three growth forms.

The results referred to above can be used to formulate a general law for the development of ecosystems: If a system receives a through-flow of exergy (energy), it will move away from thermodynamic equilibrium (this part of the formulation can be found in Morowitz[25]) and select the components and the organization that yield the highest flux of useful energy through the system (maximum power principles) and the most exergy stored in the system. It is useful to include both maximum power and exergy storage in the general law, because either concept may be more useful to apply depending on the objectives and what is known in a given situation.

The relationships between all the proposed variables (goal functions) determining the ecosystem development and the three growth forms can now be set up:

| | Growth Form I | Growth Form II | Growth Form III |
|---|---|---|---|
| Exergy storage | Up | Up | Up |
| Power/through-flow | Up | Up | Up |
| Ascendency | Up | Up | Up |
| Exergy dissipation | Up | Equal | Equal |
| Retention time | Equal | Up | Up |
| Entropy | Up | Equal | Equal |
| Exergy Biomass = specific exergy | Equal | Up | Up |
| Entropy/biomass = spec. entropy prod. | Equal | Down | Down |
| Ratio indirect/direct effects | Equal | Up | Up |

H.T. Odum[5] has also introduced the concept of emergy = the cost of energy measured in solar equivalents. It has shown that a natural system has a lower emergy/exergy storage ratio than a system controlled by man. We have not yet been able to include emergy in the scheme shown above, but H.T. Odum may do this in the future. A very important application of emergy is, however, in the comparisons of results in the ecological-economic domain.

## CONCLUSIONS

A consistent pattern of ecosystem theories has been formed as a result of join effort from many system ecologists. The pattern presented above will probably imply further progress in system ecology. This is because rules can be explained by the use of the pattern which will give a better understanding of ecosystem behavior in the future. Many rules in ecology are "lonely island" and have today no linkages to an Ecosystem theory. Linkages between rules and a theory will facilitate the deduction and prediction of rules in the future, which can explain our ecological observations.

| | | |
|---|---|---|
| Received: | December | 12, 2000 |
| Revised: | February | 2, 2001 |
| Accepted: | February | 3, 2001 |
| Published: | April | 4, 2001 |
